# An Assessment of Quality of Life in Patients With Asthma Through Physical, Emotional, Social, and Occupational Aspects. A Cross-Sectional Study

**DOI:** 10.3389/fpubh.2022.883784

**Published:** 2022-09-01

**Authors:** Zelal Kharaba, Emilie Feghali, Farah El Husseini, Hala Sacre, Carla Abou Selwan, Sylvia Saadeh, Souheil Hallit, Feras Jirjees, Hala AlObaidi, Pascale Salameh, Diana Malaeb

**Affiliations:** ^1^Department of Clinical Sciences, College of Pharmacy, Al-Ain University of Science and Technology, Abu Dhabi, United Arab Emirates; ^2^Al Ain University Health and Biomedical Research Center (HBRC), Al Ain University, Abu Dhabi, United Arab Emirates; ^3^School of Pharmacy, Lebanese International University, Beirut, Lebanon; ^4^INSPECT-LB: Institut National de Santé Publique, Epidemiologie Clinique et Toxicologie, Beirut, Lebanon; ^5^Life Sciences and Health Department, Paris-Est University, Paris, France; ^6^Health and Sciences Department, American University of Health and Sciences, Beirut, Lebanon; ^7^School of Medicine and Medical Sciences, Holy Spirit University of Kaslik, Jounieh, Lebanon; ^8^Psychology Department, College of Humanities, Effat University, Jeddah, Saudi Arabia; ^9^Research Department, Psychiatric Hospital of the Cross, Jal El Dib, Lebanon; ^10^College of Pharmacy, University of Sharjah, Sharjah, United Arab Emirates; ^11^College of Pharmacy and Health Sciences, Ajman University, Ajman, United Arab Emirates; ^12^School of Medicine, Lebanese American University, Byblos, Lebanon; ^13^Department of Primary Care and Population Health, University of Nicosia Medical School, Nicosia, Cyprus; ^14^Faculty of Pharmacy, Lebanese University, Hadat, Lebanon; ^15^College of Pharmacy, Gulf Medical University, Ajman, United Arab Emirates

**Keywords:** asthma, quality of life, satisfaction, occupational aspects, patients

## Abstract

**Background:**

Asthma is a prevalent hyperactive airway disease with physical and emotional impact. Severe asthma is associated with considerable health-related quality of life (HRQoL). The aim of this study is to assess the quality of life through physical, emotional, social and occupational aspects and evaluate the factors affecting HRQoL in patients with asthma.

**Methods:**

This is a cross-sectional multicenter study conducted on adult asthmatic patients enrolled from community pharmacies across different Lebanese geographic areas.

**Results:**

Having wheezing sometimes and most of the time (Beta = −0.144 and −0.552), experiencing anxiety sometimes and most of the time (Beta = −0.205 and −0.573), encountering sleep problems sometimes and most of the time (Beta = −0.270 and −0.553), having previous chest discomfort sometimes and most of the time (Beta = −0.421 and −0.713), and having depression most of the times (Beta = −0.415) were associated with higher lower quality of life scores. On the other side, holding a secondary level of education was associated with a higher quality of life score (Beta = 0.192).

**Conclusion:**

This study highlights that asthma affects adults' quality of life through social, emotional, physical, and occupational impacts. Improved follow-up and patient education may be essential in the future to stop disease progression and achieve ideal therapeutic outcomes.

## Introduction

Asthma is a prevalent non-communicable disease identified by chronic airway inflammation affecting children and adults worldwide (1). Differential symptoms are wheezing, dyspnea, chest discomfort, and persistent cough in addition to airflow limitation, especially at night and in the early morning. The pattern and intensity of the symptoms and airflow limitation vary over time, with exercise, allergen, exposure to irritants, weather changes, and respiratory infections, leading to exacerbation of asthma (2). Although asthma cannot be cured, exacerbations can be prevented by adequate patient counseling and proper management (3). Since it is a chronic condition, patients must utilize medications, adhere to treatment recommendations, and follow the written action for the self-control of asthma.

Even though clinical and physiological variables are used to assess asthma, they may not be enough to assess the patient's interpretation of their state of health. Thus, quality of life (QoL) is a significant endpoint as it reflects the impact of the disease from the patient's perception. Improper asthma management can have a substantial effect on the QoL, including physical, emotional, occupational, and social impacts, where the symptoms differ from one patient to another (4, 5). QoL is explained as the perception that patients have of their position in life in relation to their aims, expectations, concerns, and standards (6). The patient's wellbeing is the standard clinical outcome to assess QoL and prevent morbidity from uncontrolled disease (7).

In asthma, QoL is assessed by using the Mini asthma quality of life questionnaire (Mini AQLQ) (8); it is affected by the frequency of exacerbation, manifested through influence on daily work, deteriorated school performance, reduced social and other activities (9, 10). Other asthma-related factors that negatively affect the patient's QoL are not fully understood, and must be identified and appropriately assessed to improve the QoL (11–15). Female gender, older age, obesity, comorbid diseases such as depression, are prognostic factors associated with poor QoL (11, 16, 17). Poor QoL in asthmatic patients is associated with detrimental consequences resulting in a high prevalence of behavioral and emotional difficulties, depression, and poor academic performance (18). Moreover, avoiding triggers of asthma, and enhancing patient QoL, are effective measures to reduce morbidity and mortality (1).

In Lebanon, asthma treatment in adults falls far short of the goals specified in the international asthma guidelines, similarly to many other countries around the world. This inadequate control of the illness is associated with disease progression and poor QoL (19). In Lebanon, several studies were conducted on asthmatic patients that tackled the preschool asthma risk factor scale, evaluated association between different factors and both wheezing and asthma development, assessed asthma control, and evaluated the influence of diet and obesity on asthma (20–25). However, data on patients' QoL is still scarce, particularly among adults (26). Therefore, the purpose of this study is to assess the effect of asthma on physical, emotional, social, and occupational aspects of QoL among Lebanese adult asthmatic patients.

## Methods

### Study Design

A cross-sectional multi-centered study was conducted between February and May 2019. Six Lebanese community pharmacies from all Lebanese districts gave consent to participate in the study from a total of 13 contacted pharmacies. An online software was used to randomly select different community pharmacies using the list of pharmacies provided by the Lebanese Order of Pharmacists (OPL).

### Inclusion and Exclusion Criteria

Patients enrolled in the study were between 18 and 65 years, diagnosed by a physician to have asthma according to GINA criteria (27), and taking metered dose inhaler or dry powder inhaler for at least the past year. Patients excluded were those who could not fill the questionnaire appropriately because of decreased mental alertness or cognitive function (cognitive disorders, sedated patients, Alzheimer's disease, etc.).

### Ethical Aspect

The Institutional Review Board of the Psychiatric Hospital of the Cross approved the study and written informed consent was acquired from all participants before study enrolment. In addition, the study followed the oxford equator guidelines for cross-sectional multi-centered studies.

### Tools and Procedures

Data collection was carried out using a standardized structured questionnaire prepared in Arabic, the native language in Lebanon. A forward and backward translation was conducted for the Mini-AQLQ scale. One translator was in charge of translating the scales from English to Arabic, and another one was involved in the translation from Arabic back to English. Discrepancies between the original and translated English versions were resolved by consensus. It was administered *via* face-to-face interviews by trained researchers to ensure a higher quality of data collection. The first part of the questionnaire included patient demographics, such as gender, age, marital status, educational level (illiterate/primary, secondary, and higher education) and socioeconomic status. The second part assessed the QoL of asthmatic patients using the Mini AQLQ, a validated tool with good reliability since it is discriminates patients with different levels of impairment and validity as it measures asthma-specific quality of life (28).

This short version of the original Asthma Quality of Life Questionnaire requires a shorter time to complete (29) and includes 15 items distributed over four domains: symptoms (5 items), activity limitation (4 items), emotional function (3 items), and environmental stimuli (3 items). The symptoms field covered shortness of breath, cough, chest heaviness, sleep pattern, and wheezing. The activity limitation part assessed occupational, social, moderate, and strenuous activities. The three items evaluated by the emotional function were frustration from asthma, fear of not having the asthma medications, and concern about asthma. The fourth domain covered environmental stimuli, i.e., dust in the environment, cigarette smoking, and air pollution. The responses were scored on two 7-point Likert scales, from “all of the time” to “none of the time,” and from “severely limited” to “not limited at all.” For each item, a lower score indicated higher limitation. For each domain, the mean score was calculated by adding the answers and dividing the total by the number of questions, with scores below 6 indicating that asthma had an impact on the QoL (8). The total score was computed by summing all the answers and dividing it by the total number of questions. In our study, the reliability analysis showed a Cronbach's alpha of 0.904 for the total score, 0.717 for the symptoms' domain, 0.663 for the activity limitation domain, 0.751 for emotional function, and 0.815 for environmental stimuli.

### Sample Size Calculation

A minimum sample of 74 participants was calculated by The Epi Info software version 7.2 (population survey) to ensure a confidence level of 95%, based on a 66.7% expected frequency of anxiety in asthmatic adults and an odds ratio of 9 in the lack of similar studies in Lebanon. This study accounted for a possible non-response rate for this reason, oversampling is needed (30).

### Data Entry and Statistical Analysis

Using Statistical Package for Social Sciences version 21.0, statistical analysis was performed. Descriptive statistics were used for patients' characteristics, with frequencies and percentages for categorical variables and means ± standard deviations for continuous variables. Bivariate associations assessed through the Pearson correlation analysis for the continuous variables along with the HRQol score, and the Student *t*-test and ANOVA F tests were used for categorical variables with two or more levels, respectively. Multivariable linear regression using the Forward method was done for patient's QoL score assessment as the dependent variable, using variables that showed a *p* < 0.2 in the bivariate analysis. All reported *p*-values are two-sided, with alpha set at a significance level of 0.05.

## Results

### Socio-Demographic Characteristics

Data was collected from 200 participants out of whom 172 were enrolled in the study as the others had mental disorders and thus were excluded. The mean (±SD) age was 31.79 ± 20.92 years, with the majority being females 96/172 (55.8%), single 117/172 (68.0%), and with secondary level of education 86/172 (50.0%). 69/172 (46.3%) of families had a medium monthly income.

### Symptoms

Patients reported frequently a discomfort affecting performance of difficult tasks (49.4%), discomfort due to coughing (48.3%) and the need to avoid going out due to weather changes (47.1%). Other asthma related symptoms reported less frequently are summarized in Table 1.

**Table 1:** Demographic characteristics of study participants.

**Characteristic *N* = 172**	**Frequency (%)**
**Age mean (±SD)**	31.79 ± 20.92 years
Male gender	76 (44.2%)
Female gender	96 (55.8%)
**Marital status**	
Single/widowed/divorced	117 (68.0%)
Married	55 (32%)
**Education level**	
Illiterate/primary	59 (34.4%)
Secondary	86 (50.0%)
Higher education	27 (15.7%)
**Monthly income**	
Low	23 (15.4%)
Medium	69 (46.3%)
High	57 (38.3%)
Quality of life score mean (±standard deviation)	4.65 (±1.22)
**Quality of life based upon mini-AQLQ**	
Good	17 (9.9%)
Poor	155 (90.1%)
House crowding index (number of people living at home/number of rooms)	0.79 (±0.28)
**Previous chest discomfort**	
Never/rarely	87 (50.6%)
Few times/sometimes/quite sometimes	50 (29.1%)
Most of the time/all of the time	35 (20.3%)
**Previous wheezing experience**	
Never/rarely	86 (50.0%)
Few times/sometimes/quite sometimes	72 (41.9%)
Most of the time/all of the time	14 (8.1%)
**Depression due to asthma**	
Never/rarely	116 (67.4%)
Few times/sometimes/quite sometimes	45 (26.2%)
Most of the time/all of the time	11 (6.4%)
**Feeling discomfort due to coughing**	
Never/rarely	52 (30.2%)
Few times/sometimes/quite sometimes	83 (48.3%)
Most of the time/all of the time	37 (21.5%)
**Feeling anxious due to asthma**	
Never/rarely	97 (56.4%)
Few times/sometimes/quite sometimes	61 (35.5%)
Most of the time/all of the time	14 (8.1%)
**Discomfort to avoid difficult tasks**	
Never/rarely	53 (30.8%)
Few times/sometimes/quite sometimes	85 (49.4%)
Most of the time/all of the time	34 (19.8%)
**Discomfort or feeling that one should avoid going out due to weather changes**	
Never/rarely	59 (34.3%)
Few times/sometimes/quite sometimes	81 (47.1%)
Most of the time/all of the time	32 (18.6%)
**Difficulty sleeping due to asthma**	
Never/Rarely	75 (43.6%)
Few times/sometimes/quite sometimes	69 (40.1%)
Most of the time/all of the time	28 (16.3%)

### Quality of Life Domain Assessment

**Figure 1:**
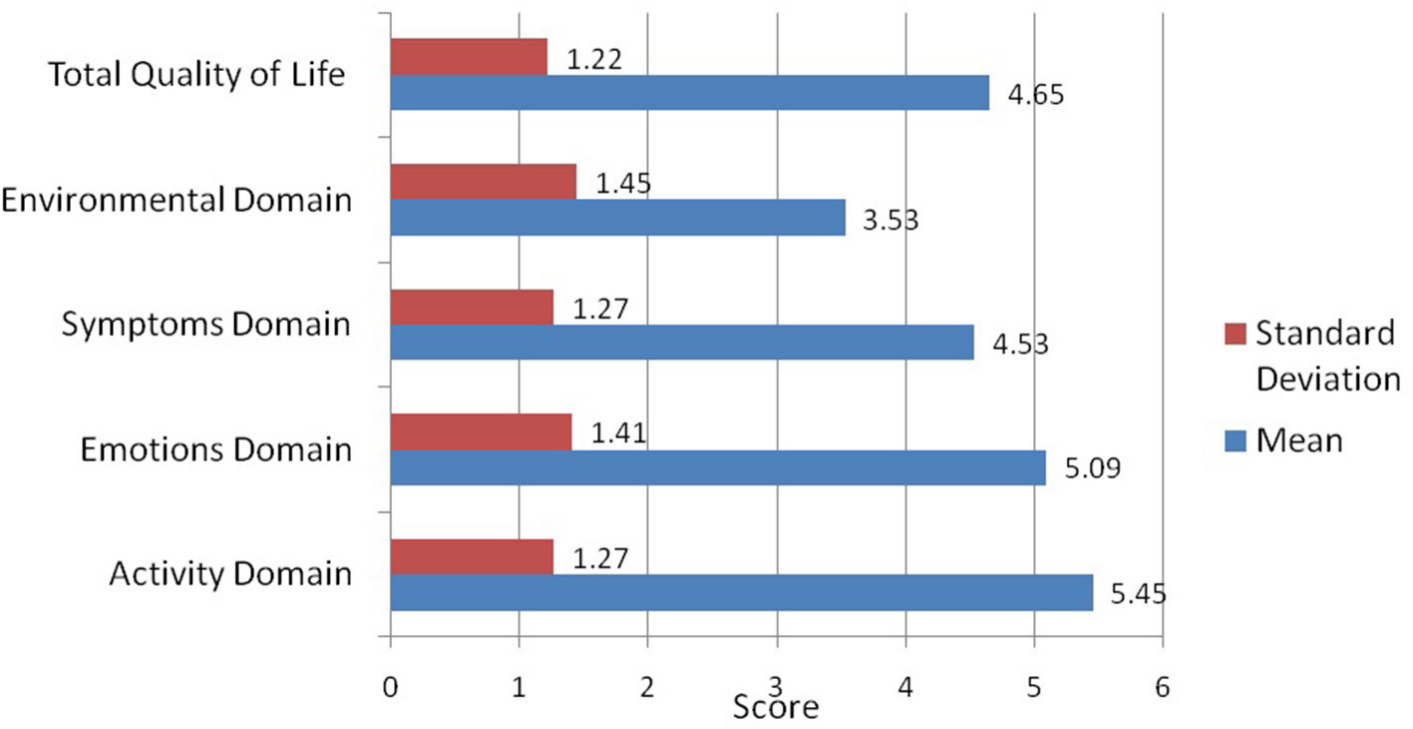
Means and standard deviation of the Quality of life scale and subscales scores.

Based upon the Mini AQLQ, the majority of patients (90.1%) had poor QoL. The mean QoL scores (±standard deviation) for the four domains of the Mini AQLQ (Figure 1) were 3.53 ± 1.45 for the environmental stimuli, 4.53 ± 1.27 for the symptoms, 5.09 ± 1.41 for the emotional function, and 5.45 ± 1.27 for the activity limitation. The mean total score of the mini-AQLQ was 4.65 ± 1.22.

### Bivariate Analysis

A meaningfully higher mean QoL score was found in males vs. females (4.88 vs. 4.50; *p* = 0.020), non-married vs. married (4.89 vs. 4.20; *p* < 0.001), high income vs. low income (4.89 vs. 4.16; *p* = 0.032) and secondary and University vs. lower levels of education (4.87 and 4.76 vs. 4.35; *p* = 0.02), and in those who had never have difficulty sleeping due to asthma (5.39 vs. 3.56; *p* < 0.001) or never had depression (5.11 vs. 2.63; *p* < 0.001) vs. most of the time. Similar results were found for feeling anxious, wheezing experience, discomfort due to coughing, discomfort related to difficult tasks and going out during weather changes. Bivariate analysis for factors associated with QoL score is summarized in Table 2. However, the results highlight that higher age was significantly associated with minor QoL score (r = −0.354, *n* = 172, *p* < 0.001) which is not illustrated in the table.

**Table 2:** Bivariate analysis for factors associated with quality of life score.

	***N* = 172**	**Quality of life score** **Mean ±Standard deviation**	***P*-value**	**Statistical test used**
**Gender**	Male	4.88 ± 0.99	0.020	Student *T*-test
	Female	4.50 ± 1.19		
**Marital status**	Single/widowed/divorced	4.89 ± 0.94	<0.001	Student *T*-test
	Married	4.20 ± 1.31		
**Educational level**	Illiterate/primary	4.35 ± 1.18	0.020	Analysis of variance
	Secondary	4.87 ± 1.08		
	Higher education	4.76 ± 0.97		
**Monthly income**	Low	4.16 ± 1.30	0.032	Analysis of variance
	Medium	4.62 ± 1.25		
	High	4.89 ± 0.84		
**Difficulty Sleeping due to asthma**	Never/rarely	5.39 ± 0.77	<0.001	Analysis of variance
	Few times/sometimes/quite sometimes	4.34 ± 0.91		
	Most of the time/all of the time	3.56 ± 1.11		
**Depression due to asthma**	Never/rarely	5.11 ± 0.88	<0.001	Analysis of variance
	Few times/sometimes/quite sometimes	4.05 ± 0.91		
	Most of the time/all of the time	2.63 ± 0.68		
**Previous chest discomfort**	Never/rarely	5.33 ± 0.65	<0.001	Analysis of variance
	Few times/sometimes/quite sometimes	4.37 ± 1.02		
	Most of the time/all of the time	3.47 ± 0.99		
**Feeling anxious due to asthma**	Never/rarely	5.27 ± 0.77	<0.001	Analysis of variance
	Few times/sometimes/quite sometimes	4.12 ± 0.91		
	Most of the time/all of the time	2.94 ± 0.95		
**Previous wheezing experience**	Never/rarely	5.26 ± 0.84	<0.001	Analysis of variance
	Few times/sometimes/quite sometimes	4.28 ± 0.96		
	Most of the time/all of the time	3.07 ± 0.96		
**Discomfort due to coughing**	Never/rarely	5.52 ± 0.80	<0.001	Analysis of variance
	Few times/sometimes/quite sometimes	4.61 ± 0.91		
	Most of the time/all of the time	3.61 ± 0.97		
**Discomfort to avoid difficult tasks**	Never/rarely	5.57 ± 0.67	<0.001	Analysis of variance
	Few times/sometimes/quite sometimes	4.68 ± 0.83		
	Most of the time/all of the time	3.27 ± 0.85		
**Discomfort to avoid going out due to weather changes**	Never/rarely	5.54 ± 0.70	<0.001	Analysis of variance
	Few times/sometimes/quite sometimes	4.61 ± 0.82		
	Most of the time/all of the time	3.24 ± 0.84		

### Multivariable Analysis

The results of a first linear regression, taking the quality of life score as the dependent variable and several other factors as independent variables, showed that holding a secondary level of education was associated with higher QoL scores. However, participants who had wheezing, anxiety, sleeping problems, chest discomfort, and discomfort to avoid going out, discomfort due to coughing, and avoidance of tasks performance on a frequency of “sometimes” and “most of the times” compared to “never” had lower QoL scores shown in Table 3. Furthermore, participants who had depression “most of the time” compared to “never,” and aging were significantly associated with lower QoL scores.

**Table 3:** Linear regression taking quality of life score as the dependent variable and wheezing, anxiety, insomnia, chest discomfort, depression, asthma knowledge score, and monthly income as the independent variables.

**Variables**	**Unstandardized beta**	**Standardized beta**	**95% confidence interval**		***P*-value**
			**Lower**	**Upper**	
Age	−0.640	−0.355	−0.896	−0.383	<0.001
Educational level (illiterate reference)					
Secondary education level	0.192	0.063	0.020	0.364	0.029
Wheezing (never reference)					
Few/some/quite-times	−0.144	−0.603	−0.296	0.007	0.062
Most and all of the time	−0.552	−0.137	−0.818	−0.285	<0.001
Anxiety (never reference)					
Few/some/quite-times	−0.205	−0.087	−0.370	−0.039	0.016
Most and all of the time	−0.573	−0.137	−0.854	−0.292	<0.001
Difficulty sleeping due to asthma (never reference)					
Few/some/quite-times	−0.270	−0.117	−0.429	−0.112	0.01
Most and all of the times	−0.533	−0.175	−0.751	−0.315	<0.001
Previous chest discomfort (never reference)					
Few/some/quite-times	−0.421	0.080	−0.578	−0.263	<0.001
Most and all of the times	−0.713	0.103	−0.916	−0.510	<0.001
Depression (never reference)					
Most and all of the times	−0.415	−0.905	−0.701	−0.128	0.005
Discomfort to avoid going out due to weather changes (never reference)					
Few/some/quite-times	−0.434	−0.190	−0.593	−0.276	<0.001
Most and all of the times	−0.756	−0.267	−0.983	−0.528	<0.001
Discomfort due to coughing (never reference)					
Few/some/quite-times	−0.294	−0.129	−0.458	−0.131	<0.001
Most and all of the times	−0.570	−0.209	−0.776	−0.365	<0.001
Discomfort to avoid difficult tasks (never reference)					
Few/some/quite-times	−0.389	−0.171	−0.551	−0.288	<0.001
Most and all of the times	−0.579	−0.207	−0.818	−0.340	<0.001

## Discussion

The study findings support the negative effect of asthma on quality of life and show the need for continuous patient monitoring and QoL evaluation during the course of the disease. Holding a secondary educational level was associated with higher QoL score, whereas aging, depression, wheezing, chest discomfort, anxiety, sleeping problems, and avoiding discomfort related to going out, discomfort due to coughing, and avoidance of tasks performance were significantly associated with lower QoL.

### Wheezing and Chest Discomfort

Wheezing and chest discomfort are critical prognostic factors in asthma (31). Indeed, wheezing is a frequent and recurrent symptom of asthma that often results in disease exacerbations and limits asthma patients' normal life. Moreover, disease severity is an essential clinical factor affecting the QoL of asthmatic patients and is associated with frequent hospitalizations and unscheduled clinic visits due to asthma exacerbation which has been documented in a previous study conducted in Germany that poorly controlled asthma is associated with a lower HRQOL in adult asthma patients (32). Consequently, wheezing and chest discomfort are associated with bad quality of life as demonstrated in our study. These findings are similar to those described in a study done in Poland on 100 asthmatic patients where high QoL scores were associated with better disease control and minimal exacerbations (11). The results emphasize on the importance of early diagnosis and management of asthma related symptoms to stop the disease progression. Our findings may be explained by the fact that patients with respiratory symptoms manifested by wheezing and chest discomfort have difficulty engaging in physical activities and daily life tasks that impair the quality of life.

### Anxiety, Depression, Sleeping Problems

Our study findings demonstrate that insomnia is highly linked to poor QoL, consistent with the results of the study conducted by Luyster et al. (33). Sleep difficulties encountered in asthmatic patients are often the outcome of nocturnal awakenings, resulting from nighttime asthma symptoms, poor control of the trigger factors, and the regular need for rescue inhaler medication; all being symptoms of uncontrolled asthma (33). Poor QoL is also related to the daytime consequence of insomnia, namely fatigue, irritability, and impaired concentration (34).

Our results also support the previous findings in which greater depression and anxiety are associated with lower QoL among asthmatic patients (35, 36). Moreover, insomnia was associated with higher levels of depression and anxiety symptoms, poor asthma control, low QoL, and more frequent asthma-related health care utilization (37). The possible hypothesis is that asthmatic patients may face physical limitation due to uncontrolled disease that enhances depression, and thus, decreases the QoL. A study conducted in Pennsylvania highlighted this finding and showed that severe asthma with increased symptom burden is positively highly associated with risk for co-morbid depression (33). Leander et al. also demonstrated a statistical correlation between anxiety, depression, and the asthma symptoms, including attacks of shortness of breath after activity, that all compromise the QoL (38).

### Aging Process

Our findings show that older age was associated with low QoL scores, in line with previous literature (32, 39, 40). The underlying evidence that explains this effect is that elderly people have higher disease exacerbation mainly due to the poor adherence to treatment, greater physical activity limitation, and end-stage disease that leads to development of irreversible asthma (40).

### Quality of Life

Quality of life is influenced by many factors, related to both the sociodemographic characteristics of the patients and comorbid disease conditions (9). This study highlights that asthma significantly affects patient's QoL as the majority of patients included in the study reported poor QoL since severe asthma has a tremendous effect on the QoL, which is an essential tool for characterizing patient populations and assessing therapeutic interventions (34). It guides healthcare professionals especially when taking care of chronic and critically ill patients. Our findings can be explained by the fact that patients with severe asthma describe poor quality of life due to excessive symptoms, frequent and life-threatening attacks, increased comorbidity burden, and high pharmacological treatment requirements (34).

The majority of the individuals endorse a poor quality of life which can be explained by the fact that many participants had secondary level of education and were of the medium socioeconomic status. According to previous literature, patients of lower socioeconomic status often report poor health behaviors that may exacerbate asthma, including higher rates of current smoking, reduced consumption of fruits and vegetables, and obesity. Also, patients with lower educational level have lower socioeconomic status and may have higher exposures to indoor and outdoor allergens, and tend to be less complaint with medication, thus increasing risk for acute asthma exacerbations which impair the quality of life (41, 42).

### Limitations and Strengths

The study has some limitations. This is a cross-sectional study where the findings cannot establish a causal relationship. Moreover, this study might be also subject to selection bias as the study enrolled more females, young age participants, and single status which can influence the quality of life. The utilization of questionnaires in the general population, especially in elderly, may not always be precise and is limited by the difficulty in questions clear comprehension and influenced by recall bias which underestimates the association between different factors and quality of life. In this study, we accounted for several variables that could affect QoL; however, not all factors were accounted for as diseases, medications, and asthma severity. To remove the confounding effect of several variables, we performed a multivariate analysis; however, we could not ignore the possibility of residual confounding of the variables that we did not evaluate as cardiovascular disease, cancer, and hypertension. Accordingly, prospective studies, taking into account these limitations, are needed. The strengths of this study include the geographical distribution of participants recruited from different pharmacies across Lebanese and the use of the mini AQLQ questionnaire in the methodology, which is a validated tool utilized to assess the QoL.

## Conclusion

The study demonstrated that asthma affects patient's QoL that was assessed through physical, emotional, occupational and social negative impacts. Patient education is an important part of treatment however, to be successful, it should not only be limited to providing knowledge, but should impact behavior and lead to a consistent change in patient's behaviors. Pharmacists and patients need to cooperate together to improve an asthma care program that targets for optimizing asthma management for a symptom-free asthma disease. During counseling, pharmacists could provide patients with adequate information about the correct use of medications and associated risks of misuse, in addition to general awareness about avoiding asthma triggers to prevent exacerbations and thus improving the QoL. In conclusion, sustained efforts are needed to optimize patients' awareness on asthma disease and its management and to dispel their myths and misconception for an improved QoL.

### Implications for Practice

Health-care professionals can utilize the findings of this study to make suitable treatment strategies for asthma for the future and develop healthcare measures based on fundamental evidence. In addition to continuous medical therapy, asthmatic patients should be provided with healthcare education and psychological consultancy services. Therefore, training and psychological support should be provided to patients to ensure optimal therapeutic outcome and disease management. It is recommended that future studies assess other factors affecting QoL among asthmatic patients and methods for improving it.

## Data Availability Statement

The original contributions presented in the study are included in the article, further inquiries can be directed to the corresponding author.

## Ethics Statement

The Institutional Review Board of the Psychiatric Hospital of the Cross approved the study and written informed consent was acquired from all participants before study enrolment. In addition, the study followed the oxford equator guidelines for cross-sectional multi-centered studies.

## Author Contributions

DM, EF, and FE analyzed and interpreted the collected data from study participants. ZK, EF, DM, HS, PS, FJ, HA, and SH contributed in idea conceptualization and study design and interpretation of data. ZK, EF, DM, FE, HS, CA, SS, SH, PS, FJ, and HA were major contributors in writing the manuscript. All authors read and approved the final manuscript.

## Conflict of Interest

The authors declare that the research was conducted in the absence of any commercial or financial relationships that could be construed as a potential conflict of interest.

## Publisher's Note

All claims expressed in this article are solely those of the authors and do not necessarily represent those of their affiliated organizations, or those of the publisher, the editors and the reviewers. Any product that may be evaluated in this article, or claim that may be made by its manufacturer, is not guaranteed or endorsed by the publisher.

## References

[1] Alith MB Gazzotti MR Montealegre F Fish J Nascimento OA Jardim JR. Negative impact of asthma on patients in different age groups. J Bras Pneumol. (2015) 41:16–22. 10.1590/S1806-37132015000100003 25750670 PMC4350821

[2] Boulet LP FitzGerald JM Reddel HK. The revised 2014 GINA strategy report: opportunities for change. Curr Opin Pulm Med. (2015) 21:1–7. 10.1097/MCP.0000000000000125 25405667

[3] Prasad R Kushwaha RAS Verma S Kumar S Verma A Prakash V. A study to know the knowledge, attitude, and practices of patients of bronchial asthma. Int J Med Public Health. (2013) 3:159. 10.4103/2230-8598.118959

[4] Sadatsafavi M McTaggart-Cowan H Chen W Mark FitzGerald J. Quality of life and asthma symptom control: room for improvement in care and measurement. Value Health. (2015) 18:1043–9. 10.1016/j.jval.2015.07.008 26686789

[5] The Significant Negative Impacts on Life With Asthma in 2017. Asthma and Allergy Foundation of America (2020). Available online at: https://community.aafa.org/blog/the-significant-negative-impacts-on-life-with-asthma-in-2017

[6] Gonzalez-Barcala FJ de la Fuente-Cid R Tafalla M Nuevo J Caamaño-Isorna F. Factors associated with health-related quality of life in adults with asthma. A cross-sectional study. Multidiscip Respir Med. (2012) 7:32. 10.1186/2049-6958-7-32 23031194 PMC3489800

[7] Al-kalemji A Petersen KD Sørensen J Sherson D Thilsing T Schlünssen V. Factors influencing quality of life in asthmatics - a case-control study: a case-control study. Clin Respir J. (2013) 7:288–96. 10.1111/crj.12006 23013421

[8] Sundh J Wireklint P Hasselgren M Montgomery S Ställberg B Lisspers K. Health-related quality of life in asthma patients - a comparison of two cohorts from 2005 and 2015. Respir Med. (2017) 132:154–60. 10.1016/j.rmed.2017.10.010 29229090

[9] Ross JA Yang Y Song PXK Clark NM Baptist AP. Quality of life, health care utilization, and control in older adults with asthma. J Allergy Clin Immunol Pract. (2013) 1:157–62. 10.1016/j.jaip.2012.12.003 24565454

[10] Alpaydin AO Bora M Yorgancioglu A Coskun AS Celik P. Asthma control test and asthma quality of life questionnaire association in adults. Iran J Allergy Asthma Immunol. (2012) 11:7. 23264406

[11] Uchmanowicz B Panaszek B Uchmanowicz I Rosińczuk J. Clinical factors affecting quality of life of patients with asthma. Patient Prefer Adherence. (2016) 10:579–89. 10.2147/PPA.S103043 27143863 PMC4844459

[12] Pickard AS Yang Y Lee TA. Comparison of health-related quality of life measures in chronic obstructive pulmonary disease. Health Qual Life Outcomes. (2011) 9:26. 10.1186/1477-7525-9-26 21501522 PMC3096892

[13] Siroux V Boudier A Anto JM Cazzoletti L Accordini S Alonso J. Quality-of-life and asthma-severity in general population asthmatics: results of the ECRHS II study. Allergy. (2008) 63:547–54. 10.1111/j.1398-9995.2008.01638.x 18394129

[14] Ford ES Mannino DM Redd SC Moriarty DG Mokdad AH. Determinants of quality of life among people with asthma: findings from the behavioral risk factor surveillance system. J Asthma. (2004) 41:327–36. 10.1081/JAS-120026090 15260466

[15] Hallit S Raherison C Waked M Hallit R Layoun N Salameh P. Validation of the mini pediatric asthma quality of life questionnaire and identification of risk factors affecting quality of life among Lebanese children. J Asthma. (2019) 56:200–10. 10.1080/02770903.2018.1441417 29513606

[16] Tay TR Radhakrishna N Hore-Lacy F Smith C Hoy R Dabscheck E. Comorbidities in difficult asthma are independent risk factors for frequent exacerbations, poor control and diminished quality of life. Respirology. (2016) 21:1384–90. 10.1111/resp.12838 27363539

[17] Grammer LC Weiss KB Pedicano JB Kimmel LG Curtis LM Catrambone CD. Obesity and asthma morbidity in a community-based adult cohort in a large urban area: the chicago initiative to raise asthma health equity (CHIRAH). J Asthma. (2010) 47:491–5. 10.3109/02770901003801980 20560823 PMC3509187

[18] Al-khateeb AJ Al khateeb JM. Research on psychosocial aspects of asthma in the Arab world: a literature review. Multidiscip Respir Med. (2015) 10:1–9. 10.1186/s40248-015-0011-6 25905019 PMC4405861

[19] Bahous J Soriano JB. Asthma control in Lebanon the asthma insights and reality in Lebanon. J Med Liban. (2010) 58:204–9. 21409942

[20] Hallit S Rahme C Sacre H Waked M Salemeh P. The Preschool Asthma Risk Factors Scale: a predictive tool for asthma and respiratory symptoms among preschool children in Lebanon. Allergol Immunopathol. (2021) 49:38–46. 10.15586/aei.v49i4.97 34224217

[21] Hallit S Raherison C Waked M Salameh P. Association between caregiver exposure to toxics during pregnancy and childhood-onset asthma: a case-control study. Iran J Allergy Asthma Immunol. (2017) 16:14. 29338155

[22] Hallit S Salameh P. Exposure to toxics during pregnancy and childhood and asthma in children: a pilot study. J Epidemiol Glob Health. (2017) 7:147–54. 10.1016/j.jegh.2017.04.004 28756822 PMC7320455

[23] Hallit S Raherison C Abou Abdallah R Hallit R Salameh P. Correlation of types of food and asthma diagnosis in childhood: a case-control study. J Asthma. (2018) 55:966–74. 10.1080/02770903.2017.1379535 28925766

[24] Hallit S Raherison C Waked M Salameh P. Validation of asthma control questionnaire and risk factors affecting uncontrolled asthma among the Lebanese children's population. Respir Med. (2017) 122:51–7. 10.1016/j.rmed.2016.11.018 27993291

[25] Malaeb D Hallit S Sacre H Rahme C Malaeb B Hallit R. Preconception exposure to over-the-counter medications and antibiotics and the risk of childhood asthma in Lebanon: a cross-sectional study. Allergol Immunopathol. (2021) 49:104–112. 10.15586/aei.v49i2.46 33641301

[26] Akiki Z Saadeh D Farah R Hallit S Sacre H Hosseini H. Asthma prevalence and associated factors among lebanese adults: the first national survey. BMC Pulm Med. (2021) 21:162. 10.1186/s12890-021-01529-z 33985479 PMC8120928

[27] Global Strategy For Asthma Management And Prevention. GINA_WR_2006.qxp:GINA_WR_2006.qxp.:110.

[28] Hossny E Caraballo L Casale T El-Gamal Y Rosenwasser L. Severe asthma and quality of life. World Allergy Organ J. (2017) 10:28. 10.1186/s40413-017-0159-y 28855973 PMC5563897

[29] Juniper EF Guyatt GH Cox F m Ferrie PJ King DR. Development and validation of the Mini Asthma Quality of Life Questionnaire. Eur Respir J. (1999) 14:32. 10.1034/j.1399-3003.1999.14a08.x 10489826

[30] Geraldo José Cunha Â Zbonik Mendes A Dias Wanderley de Carvalho F Aparecida Ribeiro de Paula M Gonçalves Brasil T. The impact of asthma on quality of life and anxiety: a pilot study. J Asthma. (2019) 56:680–5. 10.1080/02770903.2018.1486854 29969927

[31] Malaeb D Hallit S Sacre H Hallit R Salameh P. Factors associated with wheezing among Lebanese children: results of a cross-sectional study. Allergol Immunopathol. (2020) 48:523–9. 10.1016/j.aller.2020.02.003 32402625

[32] Böhmer MM Brandl M Brandstetter S Finger T Fischer W Pfeifer M. Factors associated with generic health-related quality of life in adult asthma patients in Germany: cross-sectional study. J Asthma. (2016) 54:325–34. 10.1080/02770903.2016.1206563 27624747

[33] Luyster FS Strollo PJ Holguin F Castro M Dunican EM Fahy J. Association between insomnia and asthma burden in the severe asthma research program (SARP) III. Chest. (2016) 150:1242–50. 10.1016/j.chest.2016.09.020 27720882 PMC5310183

[34] Sundbom F Lindberg E Bjerg A Forsberg B Franklin K Gunnbjörnsdottir M. Asthma symptoms and nasal congestion as independent risk factors for insomnia in a general population: results from the GA(2)LEN survey. Allergy. (2013) 68:213–9. 10.1111/all.12079 23176562

[35] Goral A Lipsitz JD Muhsen K Gross R. Depressive symptoms, risk factors and sleep in asthma: results from a national Israeli health survey. Gen Hosp Psychiatry. (2012) 34:17–23. 10.1016/j.genhosppsych.2011.09.007 22018770

[36] Luyster FS Teodorescu M Bleecker E Busse W Calhoun W Castro M. Sleep quality and asthma control and quality of life in non-severe and severe asthma. Sleep Breath Schlaf Atm. (2012) 16:1129–37. 10.1007/s11325-011-0616-8 22102290 PMC3617495

[37] Sundbom F Malinovschi A Lindberg E Alving K Janson C. Effects of poor asthma control, insomnia, anxiety and depression on quality of life in young asthmatics. J Asthma. (2016) 53:398–403. 10.3109/02770903.2015.1126846 26666333

[38] Leander M Lampa E Rask-Andersen A Franklin K Gislason T Oudin A. Impact of anxiety and depression on respiratory symptoms. Respir Med. (2014) 108:1594–600. 10.1016/j.rmed.2014.09.007 25282543

[39] Ponte EV Petroni J Ramos DCB Pimentel L Freitas DN Cruz AA. Perception of asthma control in asthma patients. J Bras Pneumol Publicacao. (2007) 33:635–40. 10.1590/S1806-37132007000600005 18200362

[40] Uchmanowicz I Uchmanowicz B Panaszek B Rosińczuk J. Sociodemographic factors affecting the quality of life of patients with asthma. Patient Prefer Adherence. (2016) 345. 10.2147/PPA.S101898 27051276 PMC4807939

[41] Barr RG Somers SC Speizer FE Camargo CA. Patient factors and medication guideline adherence among older women with asthma. Arch Intern Med. (2002) 162:8. 10.1001/archinte.162.15.1761 12153380

[42] Laurent O Filleul L Havard S Deguen S Declercq C Bard D. Asthma attacks and deprivation: gradients in use of mobile emergency medical services. J Epidemiol Community Health. (2008) 62:1014–6. 10.1136/jech.2007.064220 18854507

